# Why I Cannot Find the Prostate? Behind the Subjectivity of Rectal Exam

**DOI:** 10.5402/2012/456821

**Published:** 2012-02-15

**Authors:** Dmitry Koulikov, Ariel Mamber, Alon Fridmans, Wael Abu Arafeh, Ofer Z. Shenfeld

**Affiliations:** Department of Urology, Shaare Zedek Medical Center, The Hebrew University of Jerusalem, School of Medicine, P.O. Box 3235, Jerusalem 91031, Israel

## Abstract

*Background*. Most physicians use digital rectal examination (DRE) to help detect prostate cancer and to estimate the prostates' size. The accuracy of DRE is known to be limited. We evaluate the ability of doctors to palpate the whole prostate with DRE. *Methods*. At time of transrectal ultrasound (TRUS) the distances from the anus to the apex and base of prostates were measured. The TRUS's distances were compared to the mean index finger length of our clinic doctors. *Results*. The ability of the urologist to reach and examine the apex, half, three quarters and the whole prostate was in 93.7%, 66.3%, 23.2% and 3.2% of cases respectively. *Conclusions*. In most cases it was impossible to palpate the whole prostate. Anatomical location and volume of the examined prostate, as well as the length of his own index finger limit DRE and allow the examination of only a small portion of the prostate.

## 1. Introduction

Digital rectal examination (DRE) is widely used in medicine. A large number of physicians perform it to estimate the prostates' size and/or for early detection of prostate cancer.

It is well accepted that DRE is a subjective measure and has a high interobserver variability when estimating prostate size. It has been reported that DRE poorly predicted actual prostate size compared to transrectal ultrasound (TRUS) [1]. Others compared radical retropubic prostatectomy specimen weights with prostate weight estimates using TRUS and DRE and found that DRE correlated poorly with prostate weight and that TRUS was superior to DRE [2].

In the prostate specific antigen (PSA) era the role of DRE in early detection of prostate cancer is not clear. This role was evaluated in a number of large trials [3, 4]. The cancer detection rate for DRE was 3.2% and positive predictive value was 21%, PSA or a combination of DRE and PSA were superior to DRE alone for the diagnosis prostate cancer [3]. The positive predictive value and sensitivity of DRE were strongly dependent on PSA level and DRE predicted cancer poorly in patients with low PSA values [4]. Some have suggested that DRE may be unnecessary in patients with PSA values of 3.0 or less [5, 6], since in these patients one would need to perform 289 DREs to find one case of clinically significant prostate cancer.

We believe that in addition to DRE's subjectivity, there are other inherent limitations to DRE. When performing DRE, prior to reaching any conclusions, the examiner should be able to feel the whole posterior surface of the prostate. However, in clinical practice this may not always be possible. Patient morphometric variables as well as the length of the examiners index finger may impact on the accuracy of DRE. There is no data concerning these factors. One study examined the adequacy of prostate palpation when performing DRE during colonoscopies and concluded that patient positioning and obesity were affecting factors [7]. These observations prompted us to carry out our study.

In this study we tried to explore the limitations of DRE from an anatomical point of view. For this, the distances from anus to the prostate were measured in patients undergoing TRUS. These were compared to the length of index finger of urologists in our clinic. In other words, we examine the ability of urologist to palpate the whole of the prostates posterior surfaces at the time of DRE.

## 2. Materials and Methods

In a prospective fashion, we examined patients who were referred for prostatic biopsy at our outpatient clinic.

At time of TRUS the distances from the anus to the apex and base of prostates were measured using an 8-MHz biplane probe (B-K medical, 8808 probe). For this purpose the probe was marked with 0.5 cm gradients from the US transverse view crystal out to handle. All TRUS were performed with the patients in the left decubitus position with the knees pulled up to the chest. When the apex of the prostate was viewed in the transverse plain, its depth from the anal verge was noted (anal-apex distance). Then the probe was moved in the cephalic direction until the base of the prostate was visualized and a second measurement was noted (anal-base distance). Index fingers (from the top of the finger to the beginning third interphalangeal joint) of our clinic urologists were measured with a centimeter ruler.

All biopsies were done under local anesthesia using periprostatic block with 20 cc of lidocaine 1%. Systematic transrectal biopsies were obtained using a spring-loaded biopsy gun and 18 G biopsy needle. Patients with BMI 30 or more were excluded from the study.

To analyze the data we divided the prostatic surface length into 4 zones. First is the distal or apical zone that included the distal 25% of the prostate. The second is half prostate, the third zone included 75% of surface, and fourth zone was the whole prostate. The ability of the urologist to palpate all prostatic zones, with DRE was examined comparing the distances measured at the time of TRUS to the mean urologists' index finger length.

Commercial software (GraphPad Prism) was used for statistical analysis. The results were expressed as mean ± SEM or as median with range. All relationships were assessed by Pearson correlation analysis. Contingency table with Fisher's exact test was used to assess the accuracy of DRE. A level of significance (*P* value) < 0.05 was considered statistically significant.

## 3. Results and Discussion

**Figure 1:**
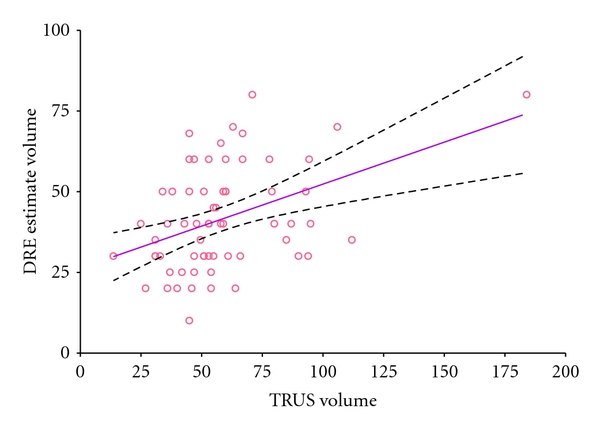
The correlation between TRUS measured volumes and DRE estimated volumes.

Between March and June 2010, ninety-five men were included in the study. The median age was 64 (range 49–82). Median PSA was 6.94 ng/mL (range 1.56–347). In Thirty-six (38%) men this was not the first biopsy session. The median number of biopsies was 12 (range 8–16). In 15 men (16%) the DRE was suspicious for prostate cancer. Median TRUS prostate volume was 53 mL (range 13.7–301) and the median DRE estimated prostate volume was 40 mL (range 10–80). The correlation between TRUS measured volumes and DRE estimated volumes was not high (Pearson *r* = 0.42) but statistically significant (*P* < 0.0006) (Figure 1).

**Figure 2:**
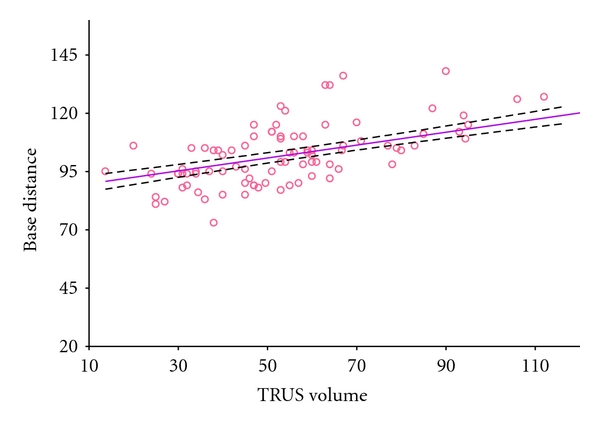
The correlation between anal-base distance and the TRUS volume of prostate.

 The median anal-apex distance was 5 cm (range 3–7.5), and anal-base distance was 10.3 cm (range 7.3–15.7). The median length of our urologists index fingers was 8.25 cm (range 7–9, *N* = 7). Thus in most cases it was impossible to palpate the whole posterior surface of the prostates. In fact, the ability of the urologist to reach and examine the apex, half prostate, three quarters, and the whole prostate was in 93.7%, 66.3%, 23.2%, and 3.2% cases, respectively. There was a good correlation between anal-base distance and the TRUS volume of prostate (Pearson *r* = 0.72, *P* < 0.001) (Figure 2).

 Twenty-nine (30.5%) cases of prostate cancer were diagnosed. The sensitivity and specificity of DRE for the diagnosis of prostate cancer were 21% and 86%, respectively. The positive predictive value of DRE was 40%. According to the Fisher's exact test the DRE was an inaccurate exam (*P* = 0.38).

This study demonstrated that in most cases it was impossible to palpate the whole posterior surface of the prostate by DRE. This is first time that distance from anal verge to prostate was recorded. Our findings may explain why DRE is a poor predictor of prostate volume and has low sensitivity for the detection prostate cancer.

### 3.1. DRE and Prostate Volume

The ability to estimate prostate volume is very important before surgical intervention, brachytherapy, benign prostatic hyperplasia management, and calculating PSA density [8–10]. According to the available literature, DRE is an inaccurate test. DRE underestimates prostate size, particularly if prostate volume is greater than 30 mL [1]. In our study a strong correlation between the anal-base distance and prostate volume was demonstrated. It follows, then, that in larger prostates even more of the prostatic surface would be beyond the reach of the palpating finger making it even harder to estimate its volume. Therefore DRE volume estimations may correlate well with TRUS measured volumes in small glands. In our study we found that DRE estimated volume did not correlate well with TRUS measured volume (Pearson *r* = 0.42). Even worse correlation was reported by Loeb et al. in their large study [2]. Although, in their study they compared DRE estimated prostatic volume with the actual weight of radical prostatectomy specimens. Based on our, and others' findings, DRE is not a good predictor of actual prostatic volume though it may help distinguish small prostates from large ones and may estimate precisely prostatic volumes in patients with small glands.

### 3.2. DRE and Prostate Cancer Screening

The debate concerning prostate cancer screening is still underway. Even after the publication of the results of large trials from USA [11] and Europe [12] the question “to screen or not to screen” remains unanswered.

In the US, the Prostate, Lung, Colorectal, and Ovarian (PLCO) Cancer Screening Trial, Andriole et al. [11] reported no mortality benefit from combined screening with PSA testing and DRE over a median followup of 11 years. In the European Randomized Study of Screening for Prostate Cancer (ERSPC) trial, Schröder et al. [12] reported that PSA screening without DRE was associated with a 20% relative reduction in the death rate from prostate cancer at a median followup of 9 years, with an absolute reduction of about 7 prostate cancer deaths per 10,000 men screened. In the PLCO study DRE was used as a screening tool whereas in the ERSPC trial DRE was used only in a small portion of the patients. One may conclude then that DRE is not helpful in detection prostate cancer. On the other hand, DRE is an integral part of nomograms and predictive tools for the management of patients with prostate cancer [13–15]. And positive DRE may predict worse prognosis comparing to normal DRE. Gosselaar et al. [16], from the Rotterdam section of ERSPC, point out that potentially aggressive cancers (Gleason score 7) are more prevalent among men who have an abnormal DRE compared to normal DRE. However, Gosselaar et al, in another study [17], state that an initially suspicious DRE, after initial cancer-negative biopsy, did not influence the chance for detection of cancer or significant cancer at later repeated biopsies. Moreover, this group of investigators previously reported that an abnormal DRE was not an indication for prostate biopsy in men with PSA < 3 ng/mL [4]. Interestingly Vis et al. [6] reported in their study that up to 63% of prostate cancers were detected coincidentally and not as a result of true positive DRE. In our study DRE was an inaccurate predictor of prostate cancer (*P* = 0.38) with low sensitivity though its specificity was 86%. According to our morphometric findings, an examiner needs to take into consideration, when performing DRE, that in most cases at least part of the surface of the prostate remains out of his reach and unexamined.

Before discarding DRE one should consider that DRE is also an important part of the physical examination of patients with lower urinary tract symptoms. Perineal sensation, anal tone, and the bulbocavernous reflex are simple and straight forward methods to assess the neurological integrity of the lower urinary tract [18].

There are some limitations to our study. Firstly, we did not take into account the elastic property of human tissue. At the time of DRE one may reach deeper by applying pressure on the perineum. Also, the shape of prostate was not taken into consideration. In large prostates, that protrude more into the rectum the actual length of the prostate grows as the curve is longer than a straight line. In our study we used a straight TRUS probe to measure prostatic length therefore the actual anal-base distance may be underestimated in some cases. This, though, does not weaken our conclusions.

## 4. Conclusions

DRE is still an important and inexpensive tool for the physician. One may gain information about the prostates size and consistency as well as the neurological function of the lower urinary tract. However, the examiner has to consider factors that limit the accuracy of DRE such as the anatomical location and volume of the examined prostate, as well as the length of his own index finger. One must remember that in many patients these factors limit DRE and allow the examination of only a small portion of the prostate.

## References

[1] Roehrborn CG, Girman CJ, Rhodes T (1997). Correlation between prostate size estimated by digital rectal examination and measured by transrectal ultrasound. *Urology*.

[2] Loeb S, Han M, Roehl KA, Antenor JAV, Catalona WJ (2005). Accuracy of prostate weight estimation by digital rectal examination versus transrectal ultrasonography. *Journal of Urology*.

[3] Catalona WJ, Richie JP, Ahmann FR (1994). Comparison of digital rectal examination and serum prostate specific antigen in the early detection of prostate cancer: results of a multicenter clinical trial of 6,630 men. *Journal of Urology*.

[4] Schroder FH, van der Maas P, Beemsterboer P (1998). Evaluation of the digital rectal examination as a screening test for prostate cancer. Rotterdam section of the European randomized study of screening for prostate cancer. *Journal of the National Cancer Institute*.

[5] Vis AN, Hoedemaeker RF, Roobol M, Van Der Kwast TH, Schrder FH (2001). Tumor characteristics in screening for prostate cancer with and without rectal examination as an initial screening test at low PSA (0.0–3.9 ng/ml). *Prostate*.

[6] Vis AN, Kranse R, Roobol M, Van Der Kwast TH, Schröder FH (2002). Serendipity in detecting disease in low prostate-specific antigen ranges. *BJU International*.

[7] Marshall JB (2008). How adequate is digital rectal exam for prostate cancer screening at colonoscopy? Can adequacy be improved?. *Digestive Diseases and Sciences*.

[8] Babaian RJ, Fritsche HA, Evans RB (1990). Prostate-specific antigen and prostate gland volume: correlation and clinical application. *Journal of Clinical Laboratory Analysis*.

[9] Roehrborn CG, Chinn HKW, Fulgham PF (1986). The role of transabdominal ultrasound in the preoperative evaluation of patients with benign prostatic hypertrophy. *Journal of Urology*.

[10] Roehrborn CG, McConnell J, Bonilla J (2000). Serum prostate specific antigen is a strong predictor of future prostate growth in men with benign prostatic hyperplasia. PROSCAR long-term efficacy and safety study. *Journal of Urology*.

[11] Andriole GL, Berg CD, Crawford ED (2009). Mortality results from a randomized prostate-cancer screening trial. *The New England Journal of Medicine*.

[12] Schröder FH, Hugosson J, Roobol MJ (2009). Screening and prostate-cancer mortality in a randomized european study. *The New England Journal of Medicine*.

[13] Stephenson AJ, Scardino PT, Eastham JA (2006). Preoperative nomogram predicting the 10-year probability of prostate cancer recurrence after radical prostatectomy. *Journal of the National Cancer Institute*.

[14] Ross PL, Gerigk C, Gonen M (2002). Comparisons of nomograms and urologists’ predictions in prostate cancer. *Seminars in Urologic Oncology*.

[15] Partin AW, Kattan MW, Subong ENP (1997). Combination of prostate-specific antigen, clinical stage, and Gleason score to predict pathological stage of localized prostate cancer: a multi- institutional update. *Journal of the American Medical Association*.

[16] Gosselaar C, Roobol MJ, Roemeling S, Schröder FH (2008). The role of the digital rectal examination in subsequent screening visits in the European randomized study of screening for prostate cancer (ERSPC), Rotterdam. *European Urology*.

[17] Gosselaar C, Roobol MJ, van den Bergh RCN, Wolters T, Schröder FH (2009). Digital rectal examination and the diagnosis of prostate cancer-a study based on 8 years and three screenings within the European randomized study of screening for prostate cancer (ERSPC), Rotterdam. *European Urology*.

[18] Fowler CJ (1996). Investigation of the neurogenic bladder. *Journal of Neurology Neurosurgery and Psychiatry*.

